# Characterization of Aquaporin Z proteoliposome structure and functionality via microscopy and scattering methods

**DOI:** 10.1007/s00249-025-01790-8

**Published:** 2025-08-07

**Authors:** Zsófia Edit Szathmáry, Martin Cramer Pedersen, Alec Michels, Torsten Høybye Bak Regueira, Jacob Judas Kain Kirkensgaard

**Affiliations:** 1 Department of Food Science, University of Copenhagen, Rolighedsvej 26, 1958 Frederiksberg, Denmark; 2 Aquaporin A/S, Nymøllevej 78, 2800 Kgs. Lyngby, Denmark; 3 Niels Bohr Institute, University of Copenhagen, Universitetsparken 5, 2100 Copenhagen, Denmark

**Keywords:** Proteoliposome, Aquaporin, Electron microscopy, Small-angle X-ray scattering, Stopped flow-light scattering, Osmotic permeability

## Abstract

Aquaporins are known for their efficient water transport capabilities and have been widely studied in the past decades. However, creating a biomimetic system mirroring natural water filtration processes still poses a challenge related to performance and stability. To study the protein reconstitution and functionality, this work presents an analytical toolkit using the model system of AqpZ reconstituted phosphatidylcholine proteoliposomes. Combining findings from dynamic light scattering, cryogenic transmission electron microscopy, laser scanning confocal microscopy, stimulated emission depletion microscopy, stopped flow-light scattering and small-angle X-ray scattering provides an assessment of structural and functional characteristics of AqpZ embedding in the bilayer of liposomes. Findings of this work reveal that the incorporation of AqpZ into liposomes promotes an increase within the hydrophobic bilayer thickness as well as within the overall size of the vesicles. AqpZ, AqpZ-GFP and AqpZ-Atto594 are studied and show distinct permeability profiles. Despite all three displaying a successful structural reconstitution into the liposomes, labeled protein variants demonstrate a loss of function. A series of protein concentrations are utilized to extract quantitative information regarding the reconstitution process, revealing constant water transport per AqpZ and thus a consistent trend of increased reconstitution and permeability as a function of AqpZ concentration, as determined by stopped flow-light scattering and detailed further via global fitting of small-angle X-ray scattering data.

**Supplementary Information:**

The online version contains supplementary material available at 10.1007/s00249-025-01790-8.

## Introduction

Water scarcity is predicted to become one of the greatest threats of the century (WWAP 2022) requiring a fresh look on water purification, desalination and wastewater management (WWAP 2022; FAO 2011). Membrane filtration technologies provide an energy efficient and easily scalable way of water purification. Synthetic membranes are mostly made of organic polymers (Mulder 1991) and particularly polyamide (PA) based reverse osmosis (RO) membranes are widely used on a global scale for diverse applications (Elfil et al. 2007; Shannon et al. 2008; Wang et al. 2013). To further improve performance, membranes can be modified (1) physically by more efficient membrane packing, (2) chemically by using synthetic additives or (3) biologically by adding a natural compound such as a protein (Ulbricht 2006; Kong et al. 2011; Zhao et al. 2012). In recent years, the latter approach has been gaining significant attention, resulting in the appearance of biomimetic membranes (Tang et al. 2015). A particular group of membrane proteins that have been widely studied for doping the selective layer, are aquaporin proteins (Aqps) (Gan et al. 2017).

**Fig. 1:**
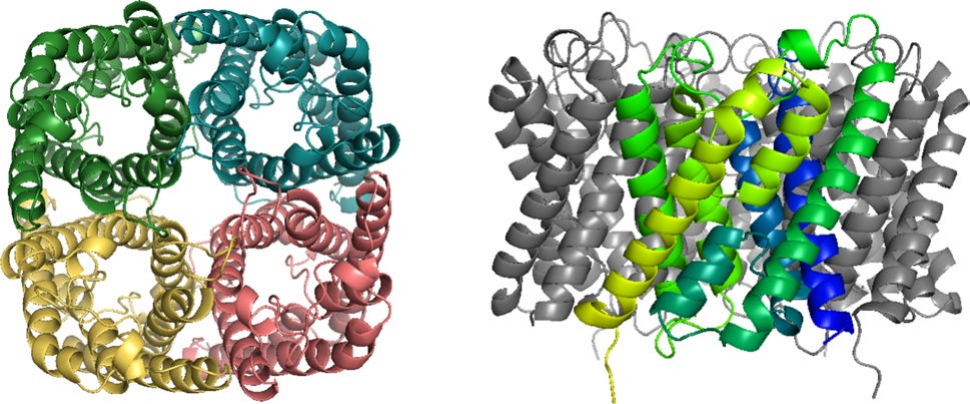
Structure of the AqpZ protein. Top and side views displaying the tetrameric AqpZ complex, where each monomer is marked with a different color and one monomer is highlighted in a gradient from blue to yellow from the N to C terminal, respectively. Models were generated using PyMol 2.5.2 with the AqpZ Protein Data Bank (PDB) structure, 2ABM (Kim et al. 2020)

In nature, Aqps are present in the phospholipid membranes of living cells and are primarily responsible for cellular water transport (Grzelakowski et al. 2015; Habel et al. 2015; Takata et al. 2004). Aqps are able to transport more than three billion water molecules per second per protein, while rejecting solutes and charged compounds (Gan et al. 2017). Crystallographic studies describe Aqps with an “hourglass model”, which refers to the protein’s ability to create a hydrophilic pore for water molecules to pass through cell membranes (Takata et al. 2004; Heymann et al. 1997; Cheng et al. 1997). Further electron microscopy studies showed that Aqps are present in the membrane as homotetramers, consisting of four identical but independently functioning water transporting pores (Fig. 1) (Takata et al. 2004; Banga 1998).

While protein crystallography serves as an important tool to learn more about the structure of membrane proteins, it has its limitations (Berthaud et al. 2012). Electron microscopy has shown a great promise in recent years, enabling scientists to resolve structural features of proteins at atomic resolution (Nakane et al. 2020; Yip et al. 2020) which has led to an impressive output of structural information for complex biophysical systems; including Aqps (Kamegawa et al. 2023; Huang et al. 2023). Small Angle Scattering (SAS) techniques provide the bridge between these static structures and an integrated, dynamic view of these complexes enabling an in depth understanding of their roles in biological systems.

By being insoluble in aqueous buffers upon membrane extraction, the polyphilic Aqps must be solubilized in detergent micelles to maintain proper folding (Agre et al. 1993; Agre et al. 2002; Bill and Hedfalk 2021). Studying these protein containing detergent micelles via small-angle X-ray scattering (SAXS) has shown to be an important step towards conformational resolution. By modeling the detergent corona, three-dimensional information can be extracted of the protein’s structure (Berthaud et al. 2012; Koutsioubas et al. 2013).

Besides detergent micelles, a wide-range of self-assemblies have been studied with the aim of protecting Aqp proteins and mimicking their natural cellular environment (Gan et al. 2017; Beales et al. 2017; Xie et al. 2013). Lipid bicelles, amphiphilic polymer assemblies, nanodiscs and styrene maleic acid lipid particles (SMALPs) have all been widely investigated for this purpose (Popot et al. 2011; Dürr et al. 2012; Bayburt and Sligar 2010; Frauenfeld et al. 2016; Lee et al. 2016). Even though a range of these structures have proven to be successful at hosting the protein, they all lack the important feature of closed compartment-based encapsulation, where there is a transmembrane potential between two separate segments of the reconstitution assembly (Ichihashi and Yomo 2014; Go and Jones 2008). Thus, in order to mimic their native structure and functionality best, said compartments should be represented in the synthetic nanocarrier system, to quasi-artificially induce a potential and osmolarity difference (Hill and Shachar-Hill 2015).

A widely studied compartment-based system is the liposome, which besides possessing a native-like bilayer (Grzelakowski et al. 2015; Bill and Hedfalk 2021; Borgnia et al. 1999), also has a distinct external and internal space, similar to biological cells, which further favors protein embedment as detailed above (Ichihashi and Yomo 2014; Go and Jones 2008). Even though the formation of liposomes with reconstituted Aqps has been widely reported in literature, proteoliposomes serve as a valuable model system for the detailed investigation of lipid–protein interactions (Gan et al. 2017; Grzelakowski et al. 2015; Beales et al. 2017; Kim et al. 2020).

Although AqpZ incorporation into proteoliposomes is not a novel approach on its own, the present work is unique in employing several independent scientific methods to gain a better understanding of the delicate system dynamics and further investigate the degree of AqpZ reconstitution within the liposomes. In order to facilitate protein incorporation and mimic the lipid heterogeneity of the cellular membrane, the selected liposome forming L-α-Phosphatidylcholine (soy PC) lipid consisted of a PC lipid mixture with different alkyl chains such as palmitic, stearic, oleic, linoleic and linolenic (Sigma. 2021). Besides resembling the natural environment of AqpZ, the selected mixture showed an ease of liposome formation via spontaneous self-assembly, while being biocompatible and widely available (Kloda et al. 2007). Overall, this study delves deep into the mechanics of AqpZ embedment into the bilayer of soy PC liposomes and combines results from (1) dynamic light scattering (DLS), (2) cryogenic transmission electron microscopy (Cryo-TEM), (3) laser scanning confocal microscopy (LSCM), stimulated emission depletion (STED) microscopy, (4) stopped flow-light scattering (SF-LS) and (5) SAXS with the aim of building an analytical toolkit, adaptable to study any other membrane protein containing nanocarrier system. Furthermore, the reconstitution of three AqpZ variants: AqpZ-His (AqpZ), AqpZ-Green Fluorescent Protein-His (AqpZ-GFP) and AqpZ-His-Atto594 NHS-ester (AqpZ-Atto594) is demonstrated into liposomes, by comparing findings from multiple analytical methods in order to critically evaluate the mechanisms of the proteoliposome system (for more information on the AqpZ variants see Experimental Section & Supplementary Information).

## Experimental

### Materials

Soy PC granules (≥ 30%) extracted from soybeans were obtained from Sigma-Aldrich (Germany). 10xPhosphate Buffer Saline (PBS) stock solution was prepared by combining 1360 mM NaCl, 26 mM KCl, 81 mM Na_2_HPO_4_ and 18 mM KH_2_PO_4_ in MilliQ water. The solution was pH adjusted to 7.4 with HCl. All chemicals for the preparation of PBS were analytical grade, obtained from Merck (Germany). NaCl powder was obtained from Sigma-Aldrich (Germany) for the preparation of a 50 mM, sterile filtered solution. Detergents, 30 w/v% N, N-Dimethyl-n-dodecylamine N-oxide (LDAO) and Octyl-beta-Glucoside (OG) powder, were obtained from Sigma-Aldrich (Germany) and Biosynth (UK), respectively. AqpZ and AqpZ-GFP proteins were produced and purified in house at Aquaporin A/S, expressed from *Escherichia coli* and purified by immobilized metal affinity chromatography (IMAC) via the His tag (Bjørkskov et al. 2017; Myers et al. 2019). AqpZ was covalently labeled post purification with the Atto594 fluorophore, obtained from Atto-Tec (Germany). The labeling was performed according to the manufacturer’s instructions at pH 8.3 to target the primary amines of the polypeptide chain and it was followed by size exclusion chromatography (SEC), to separate the labeled and unlabeled proteins as well as the free dye. The labeled protein was further characterized by sodium dodecyl sulfate–polyacrylamide gel electrophoresis (SDS-PAGE) (for results, see Supplementary Fig. 1 and Supplementary Tables 1 and 2). Proteins, with the exception of the SEC purified labeled protein, were dialyzed into a 1xPBS buffer containing 10% glycerol and 0.2 w/v% LDAO to their final concentration, which was assessed by the bicinchoninic acid assay (BCA). Glycerol (≥ 99.5%) and chloroform were obtained from Sigma-Aldrich (Germany). Atto633-PPE dye (≥ 90%) was purchased from Atto-Tec (Germany). Bio-beads SM-2 Resin was purchased from Bio-Rad (USA).

### Soy phosphatidylcholine liposome preparation and Aquaporin Z reconstitution

**Fig. 2:**
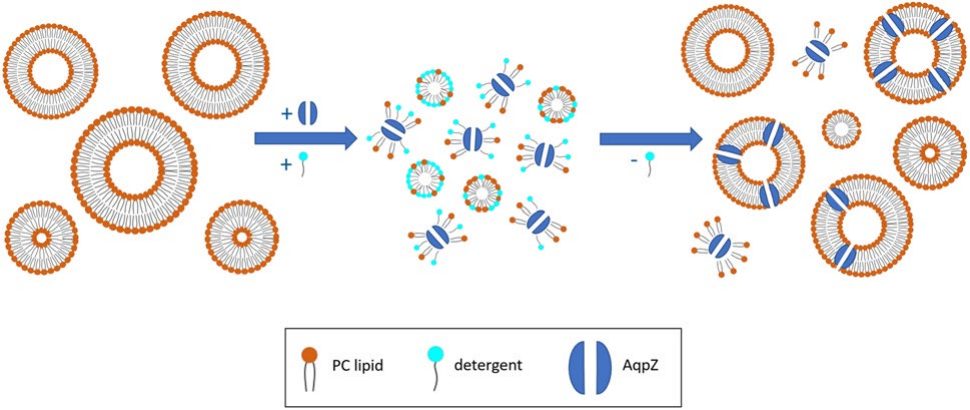
Schematics of the detergent mediated direct hydration process of AqpZ reconstitution into soy PC liposomes

AqpZ incorporated soy PC liposomes were prepared using a direct hydration method (Fig. 2). Lipids were first dissolved in a 1xPBS buffer, followed by sonication, using an ultrasonic bath (Branson, USA), yielding a final concentration of 10 mg/ml. Their average hydrodynamic diameter, Z-avg, and polydispersity index, PDI, were measured by DLS, using the Zetasizer Nano instrument (Malvern, UK). Samples were measured at 20 °C, using poly (methyl methacrylate) (PMMA) cuvettes (Sarstedt, Germany). Thereafter, liposomes were incubated in the presence of the selected AqpZ protein variant and 1.1 w/v% OG in 1xPBS for 1 h at room temperature. For quantification purposes, three protein concentrations were tested: 0.05 mg/ml, 0.1 mg/ml and 0.2 mg/ml. The final lipid concentration was kept at 7.5 mg/ml for all samples (Sharma et al. 2022; Zhao et al. 2022) yielding lipid to protein (tetramer) molar ratios of 18,713:1, 9356:1 and 4678:1 respectively for the three concentrations. Detergent was removed by a step-wise addition of Bio-beads, where 40 mg of beads were added for 1 mg detergent and samples were placed on a rocking platform shaker (VWR, USA). Complete detergent removal was ensured via overnight tilting at 4 °C. The next day, all Bio-beads were removed and reconstituted liposome samples continued to be stored at 4 °C without tilting. For Atto633-PPE labeled samples, 1.15 µg of chloroform dissolved dye was placed on the bottom of the flask and the solvent was allowed to evaporate overnight in a fumehood at room temperature, the day before starting the reconstitution process. For consistency, all samples regardless of protein addition have been treated the same way and exposed to the same conditions.

### Stopped flow-light scattering

Before performing SF-LS, samples were diluted 10 times and extruded 15 times through a 200 nm Whatman® Nuclepore™ Track-Etched Polycarbonate Membrane (Cytiva, USA) using a liposome extruder (Avestin, Canada). Extrusion was performed in order to create a more uniform sample with respect to the formed self-assemblies, which supports the comparability of results obtained from the different experimental techniques.

The Z-avg and PDI values were determined for both non-extruded and extruded liposomes using the DLS instrument specified above. Finally, the activity of reconstituted proteins was measured by means of the SF-LS technique, using a Bio-Logic SFM 300 SF-LS device (Bio-Logic Science Instruments, France) with a monochromator at 517 nm, cut-off filter at 530 nm and a µFC-08 cuvette. SF-LS is a commonly used method for the characterization of protein containing vesicles (Habel et al. 2015; Zhao et al. 2022). During an experiment, the sample is rapidly mixed with a hyperosmotic solution, resulting in the shrinkage of vesicles due to the outflux of water in an attempt to equilibrate the osmotic gradient. The speed of vesicle volume change can be directly correlated to the amount of reconstituted protein in the lipid bilayer and can be monitored in real time via light scattering (Gena et al. 2020) However, it is worth to note that vesicle rupture and reformation cannot be ruled out entirely either (Liu et al. 2022).

For every measurement 113 µl of diluted sample was mixed rapidly with the same volume of 0.5 M NaCl solution at pH 7.4. The rate of volume change for proteoliposomes was measured as a function of time, with a dead time of 10 ms and 10 individual measurements per sample from the same reaction vial. Results reported are an average of 5 measurements per sample, which were normalized with respect to the scattering intensity for the total measurement period of 0.4 s. The average rate constant, K, representing the speed of vesicle shrinkage, was determined by fitting the light scattering curves to a double exponential function. The K value serves as a direct measure of water flux through the bilayer of the proteoliposomes. Using the K value, the osmotic permeability, P_f_, was determined according to the method described by Grzelakowski et al. 2015 and the calculations presented in the Supplementary Information, in Supplementary Table 3 (Grzelakowski et al. 2015). Further, following Wachlmayr et al. 2022 single channel permeabilities, p_f_, were derived (Wachlmayr et al. 2022).

### Electron and light microscopy

Proteoliposomes were also subjected to three kinds of microscopy-based analyses: Cryo-TEM, LSCM and STED. Cryo-TEM was performed either by the Tecnai G2 TWIN 200 kV TEM equipped with a FEI High-Sensitive 4k x 4k Eagle camera (Thermo Fisher Scientific, USA), used for general screening purposes of the samples or the Glacios™ 200 kV XFEG TEM equipped with a Thermo Scientific™ Falcon 3 Direct Electron Detector (Thermo Fisher Scientific, USA) used for high resolution structural determination of the liposome bilayer at a raw pixel size of 1 Å and a defocus range of 0.5–3 µm. Samples were vitrified on lacey formvar film, enforced by silicon monoxide coating and supported by a copper mesh grids (Ted Pella Inc., USA). Grids were glow discharged using the Leica Coater ACE 200 for 60 s at 10 mA (Leica, Germany). Vitrification was performed in liquid ethane cooled by liquid nitrogen and with the use of Vitrobot Mark IV (Thermo Fisher Scientific, USA) and the following conditions: 4 °C, 100% humidity, 5 s blotting time, −8 blotting force. Samples were either loaded on the Gatan cryoholder (Thermo Fisher Scientific, USA) for analysis on the Tecnai or on the Autoloader™ (Thermo Fisher Scientific, USA) for analysis on the Glacios™. Results were processed with the ImageJ software (National Institutes of Health, USA), which included 10–15 individually captured images for each sample. For assessing the mean vesicle size in a given sample, the diameter of 20 individual liposomes was measured and averaged. While the membrane’s hydrophobic thickness was measured at 50 unique locations that were selected along different vesicles. In both cases, the Measure function of ImageJ was used. LSCM was performed by the Zeiss LSM900 microscope equipped with two standard GaAsPs detectors and an Airyscan2 detector, a 63x/1.3 objective and an AxioCam 702 mono camera using the Colibri7 LED light source (Zeiss, Germany). Samples were immobilized on a microscope slide via polylysine and were diluted 10 times. Excitation of Atto633-PPE was performed at 640 nm (red channel) and AqpZ-GFP at 475 nm (green channel), respectively. Samples were processed with the ZEN software (Zeiss Group, Germany).

STED was carried out using the Abberior STEDYCON STED system equipped with a 775 nm depletion laser (Abberior Instruments, Germany), which enabled far-red and orange super-resolution imaging. Samples were prepared on a standard microscope glass slide with a water-based mounting medium, Abberior Mount Solid Antifade (Abberior Instruments, Germany) followed by applying a cover slip and sealing with a nitrocellulose adhesive. Data deconvolution was carried out using the Huygens Professional software (Scientific Volume Imaging, the Netherlands).

### Small-angle X-ray scattering

Lastly, SAXS was performed on non-extruded samples as extrusion is known to cause material losses inducing concentration differences in the samples (Wagner and Vorauer-Uhl 2011). In this study, lab-scale SAXS was carried out using the Nano-inXider SAXS (Xenocs, France), equipped with a λ = 1.54 Å wavelength Cu K_α_ source (Rigaku-Denki, Japan) and a Pilatus detector (Dectris, Switzerland). The samples were loaded into borosilicate glass capillaries (Hilgenberg, Germany outer diameter: 1.5 mm, wall thickness: 0.01 mm, length: 80 mm) and sealed airtight. Acquisition was carried out with a 800 µm beam size, 8·10^7^ ph/s flux and 0.0028 Å^−1^ beam resolution at room temperature, for 60 min per sample. The range of scattering momentum transfer, q, was from 0.004 to 0.35 Å^−1^, with q defined by:

1 $$q=\frac{4\pi \text{sin}\theta }{\lambda }$$

where θ is half of the scattering angle. When studying liposomes, depending on the q-range the scattering curves can provide information both about the overall size and shape of the vesicles (at small q values), as well as the structure of the bilayer (at larger q values) (Varga et al. 2014). For post-measurement processing of the scattering data the XSACT software (Xenocs, France) was used where background subtraction and scaling were carried out as well as logarithmic re-binning, using standard settings. The SAXS data was analyzed using a custom model to extract information regarding the lipid bilayer, where a solution of liposomes described by a lamellar model (Nallet et al. 1993; Pabst et al. 2000) was combined with a small amount of lipid micelles, described by a core–shell model (Guinier et al. 1955). The presence of lipid aggregates in the sample had been previously verified through an independent ultracentrifugation study, followed by LSCM (for results see Supplementary Information and Supplementary Fig. 2) (Gan et al. 2017). For simplicity, these lipid aggregates were given the attributes of micelles when performing the SAXS data modeling.

**Fig. 3:**
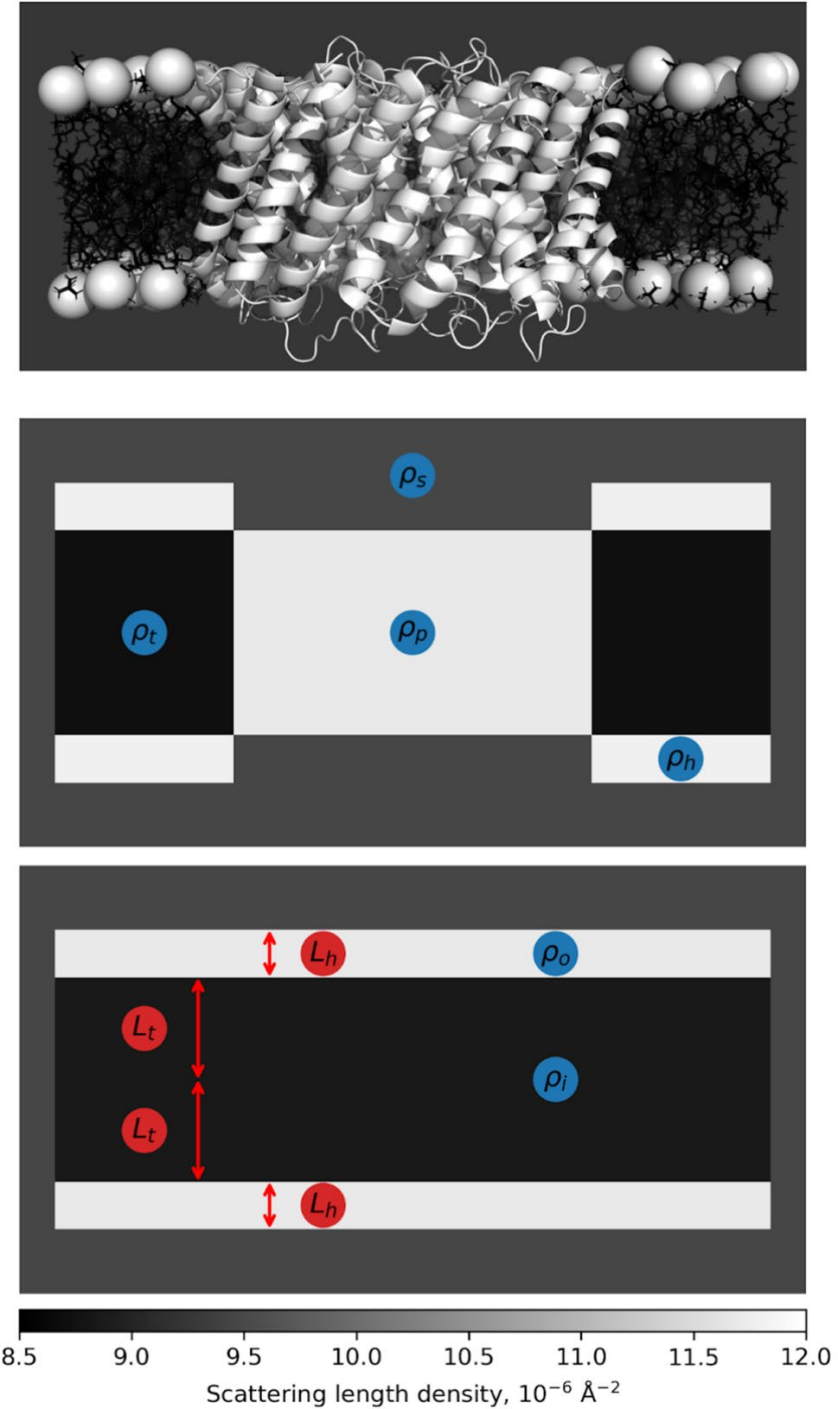
Schematics explaining the model with associated parameters and scattering properties. On top, the atomic model of AqpZ is shown, visualized using the PDB structure 2ABM (Kim et al. 2020) embedded in a phospholipid membrane, generated using CharmmGUI (Wu et al. 2015; Jo et al. 2008). In the middle, a simple geometric description of this structure is introduced. Here, the SLDs of the various components are indicated in blue. Below, the model is established for analysing the SAXS data by computing the average SLDs in the various parts of the bilayer as shown in Eqs. 2 & 3. Apart from the SLDs, once again highlighted in blue, the structural parameters are annotated in red. As indicated by the color scale in the bottom, the grayscale of the images denotes the estimate of their SLDs. The dimensions match the ones from data refinement

The model is depicted in Fig. 3 and is parameterized as follows: 1) the length of lipid alkyl tails, L_t_, 2) the length of a PC headgroup, L_h_, as well as the scattering length densities (SLDs) of 3) the solvent, ⍴_s_, 4) the lipid alkyl tails, ⍴_t_, 5) the lipid PC headgroups, ⍴_h_, and 6) AqpZ, ⍴_p_, and finally 7) the amount of AqpZ embedded in the hydrophobic part of the bilayer, represented as a volume fraction x. For simplicity it is assumed that the protein in its entirety is embedded in the hydrophobic part of the bilayer and that the space above and below an embedded protein is occupied by solvent.

This allowed to calculate the effective SLDs for the hydrophobic (inner) and hydrophilic (outer) part of the bilayer, ⍴_i_ and ⍴_o_:

2 $${\rho }_{i}=x{\rho }_{p}+\left(1-x\right){\rho }_{t}$$

3 $${\rho }_{o}=x{\rho }_{s}+\left(1-x\right){\rho }_{h}$$

The SLDs of the model were fixed at values found in the literature. Specifically, ⍴_s_ = 9.46·10^–6^ Å^−2^^(^Erko et al. 2012), ⍴_t_ = 8.7·10^–6^ Å^−2^ (Harvey et al. 2023; Ristori et al. 2005) and ⍴_h_ = 11.8·10^–6^ Å^−2^^(^Harvey et al. 2023). The SLD of AqpZ was calculated as ⍴_p_ = 11.7·10^–6^ Å^−2^, according to the AqpZ-His sequence described in the Supplementary Information and the SASSIE Contrast Calculator (Sarachan et al. 2013). The contrasts of the different components (∆⍴) in the model were set by the amount of AqpZ in the bilayer, x. Furthermore, a scale for the lamellar, C_1_, and micellar, C_2_, models was also refined as well as a constant background, B, and a term accounting for the roughness of the interfaces in the model, R (Als-Nielsen and McMorrow 2011). The final fit combined the scattering intensity of the lipid bilayer, I (q)_lam_, described by the lamellar model, where Δ⍴_o_ and Δ⍴_i_ indicate the contrasts of the outer and inner bilayer areas to the solvent,

4 $${I(q)}_{lam}=\frac{4\pi }{{q}^{4}({L}_{h}+{L}_{t})}{\left(\Delta {\rho }_{o}\left(\text{sin}\left(q\left({L}_{h}+{L}_{t}\right)\right)-\text{sin}\left(q{L}_{t}\right)\right)+\Delta {\rho }_{i}\text{sin}\left(q{L}_{t}\right)\right)}^{2}$$

and the scattering intensity of the micelles, I(q)_mc_:

5 $${I\left(q\right)}_{fin}={{C}_{1}I\left(q\right)}_{lam}{e}^{{-R}^{2}{q}^{2}}+{{C}_{2}I\left(q\right)}_{mc}{e}^{{-R}^{2}{q}^{2}}+B$$

The micellar model is simply a spherical core–shell model inheriting the SLD and structural parameters from the bilayer, presented in the Supplementary Information. A global fitting strategy was employed, where all individual datasets in a given data series were analyzed in one simultaneous fit (Tidemand et al. 2022; Barclay et al. 2022). In this process only x as well as the scale and background were varied between each dataset, the remaining parameters were identical across the analyzed datasets, allowing to refine a single model accounting for all of the SAXS data. The model was implemented and the fitting was performed using WillItFit; with its source code available from the online repository (Pedersen et al. 2013). The presented error estimates were ordinary 63.8% confidence intervals (Pedersen et al. 2014; Pawitan 2001).

## Results and discussion

### Proteoliposome formation

AqpZ incorporated soy PC liposomes were prepared by the direct hydration method described above, where highly absorbent polystyrene beads ensured the removal of detergent in a gradual manner, promoting the formation of proteoliposomes. DLS was carried out on empty, as well as AqpZ reconstituted liposomes to verify the size distribution of particles by their light scattering intensity (Before extrusion, measurements of the Z-avg and PDI values provided information on the vesicles in their native state and revealed that protein loading increased the sample polydispersity as well as the size of the vesicles. This might indicate changes within the architecture of the liposome, due to the incorporation of AqpZ, which are investigated further in the upcoming sections. After extrusion all samples showed a high degree of uniformity in relation to the liposome size and polydispersity. Table 1). While some analytical methods were sensitive to varying particle concentrations, others required a highly monodisperse population of sample particles, which consequently determined the need for liposome extrusion.

**Table 1:** Hydrodynamic diameter, Z-avg, and polydispersity index, PDI, for empty and AqpZ reconstituted liposomes at 0.05 mg/ml, 0.1 mg/ml and 0.2 mg/ml protein concentrations, respectively, and 7.5 mg/ml lipid concentration. Measurements were performed before and after extrusion through a 200 nm membrane

	Before extrusion		After extrusion	
	Z-avg (nm)	PDI	Z-avg (nm)	PDI
Empty liposomes	138.4	0.101	132.9	0.081
0.05 mg/ml AqpZ Liposomes	146.0	0.196	124.7	0.096
0.1 mg/ml AqpZ Liposomes	170.5	0.250	142.9	0.062
0.2 mg/ml AqpZ Liposomes	193.5	0.340	136.7	0.063

Before extrusion, measurements of the Z-avg and PDI values provided information on the vesicles in their native state and revealed that protein loading increased the sample polydispersity as well as the size of the vesicles. This might indicate changes within the architecture of the liposome, due to the incorporation of AqpZ, which are investigated further in the upcoming sections. After extrusion all samples showed a high degree of uniformity in relation to the liposome size and polydispersity.

### Cryogenic transmission electron microscopy

Cryo-TEM was used for two purposes in the current study: (1) to screen samples and assess their vesicle size distributions and lamellarity as well as (2) to resolve the bilayer thickness and monitor the differences in the bilayer structure for vesicles with and without reconstituted protein. For simplicity, the two sample types presented in this section are empty and 0.2 mg/ml AqpZ reconstituted liposomes. Samples were imaged in their unextruded state to get an accurate picture of the innate lipid-protein assemblies as well as due to the scaling difficulties of the extrusion step with respect to industrial production.

**Fig. 4:**
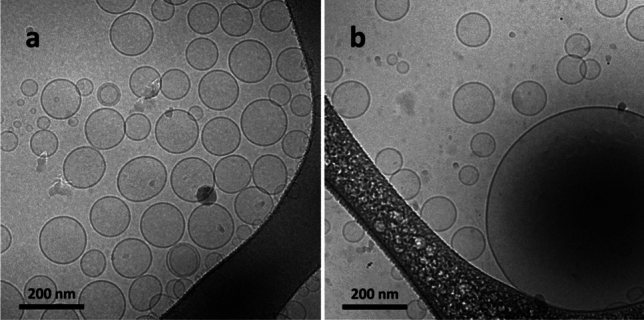
Low magnification Cryo-TEM images of **a** empty liposome and **b** 0.2 mg/ml AqpZ reconstituted liposome populations, displaying vesicle polydispersity, lamellarity and size distributions

As seen in both Table 1 and Fig. 4, empty liposomes were rather uniform in their shape and size, showing a low degree of multi-lamellarity. Cryo-TEM image analysis revealed an average vesicle diameter of 130.4 ± 15.4 nm, which was well in alignment with the DLS results presented in Table 1. The 0.2 mg/ml AqpZ reconstituted liposomes were similar in their overall size and shape to their empty counterparts, although the appearance of some larger vesicles was noted, reaching a diameter of over 500 nm. Image analysis showcased an average vesicle size of 200.2 nm for these samples, which also agree with the results in Table 1. However, due to the appearance of some significantly larger vesicles the standard deviation increased to 169.1 nm, which can be correlated to the higher PDI value observed for the DLS results of the corresponding sample.

**Fig. 5:**
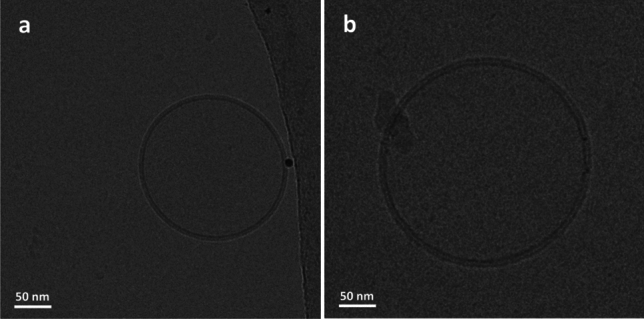
High magnification Cryo-TEM images of **a** an empty liposome and **b** a 0.2 mg/ml AqpZ reconstituted liposome

High resolution and magnification Cryo-TEM revealed information about the hydrophobic thickness of the bilayer for empty and protein loaded liposomes. As seen in Fig. 5a, for the empty liposome the bilayer appeared smooth and uniform while for the AqpZ reconstituted liposome, the bilayer had a rougher appearance (Fig. 5b). This difference is ascribed to the presence of reconstituted AqpZ, which causes changes, particularly in the hydrophobic region of the bilayer. As shown by several studies, the hydrophobic thickness of a given vesicle system plays a key role in successful membrane protein insertion (Kim et al. 2020; Habel et al. 2015; Tong et al. 2012; Schmidt et al. 2019). The closer the thickness of the hydrophobic region of the bilayer is to the hydrophobic length of the protein, the more efficiently it can insert into the membrane. However, in case of lipid-based systems, the bilayer has a degree of fluidity, enabling it to deform and match the thickness of the protein’s transmembrane region.

Conversely, it is also known that some membrane proteins undergo considerable structural rearrangements and functional changes when interacting with or embedded in lipid membranes to accommodate the physical and chemical properties of the membrane (Vitrac et al. 2015; Phillips et al. 2009). Similar effects have been observed for oligomerisation and protein complexes (Quinn and Wang 2008; Gupta et al. 2017).

When studying the hydrophobic bilayer thickness for empty and 0.2 mg/ml AqpZ reconstituted liposomes, image analysis revealed an increase from 2.8 ± 0.4 nm to 3.9 ± 0.5 nm, respectively, based on recording and averaging 50 individual hydrophobic membrane thickness values of various vesicles, using the Measure command of the ImageJ image analysis software. The former value for empty liposomes is in good agreement with the reported thickness for similar PC liposome tail regions as the currently used soy PC mix (Harvey et al. 2023). While the latter value shows an increase, corresponding to the changes within the architecture of the hydrophobic bilayer region, when accommodating the AqpZ incorporation. The appearance of a thickened bilayer and the overall diameter increase of liposomes before and after protein addition, together with the results presented in Table 1 and Fig. 4, presume that AqpZ protein insertion promotes the formation of larger liposomes with a flatter bilayer architecture.

### Laser scanning confocal microscopy

To verify the structural reconstitution of AqpZ into liposomes, LSCM was used for the allocation of the co-localized lipid dye and recombinantly expressed GFP protein tag. The Atto633-PPE lipid dye, which has affinity to the hydrophobic region of liposomes absorbs at λ_abs_ = 630 nm (orange) and emits at λ_fl_ = 651 nm (red) and it has been successfully used previously for liposome labeling and visualization (Wachlmayr et al. 2023; Antonenko et al. 2012; Saitov et al. 2022). For identifying protein reconstitution, the GFP tag was utilized which was genetically expressed on the C-terminal of the protein, one for each monomer. It absorbs at λ_abs_ = 395 nm (blue) and emits at λ_fl_ = 508 nm (green) (Laganowsky et al. 2014).

**Fig. 6:**
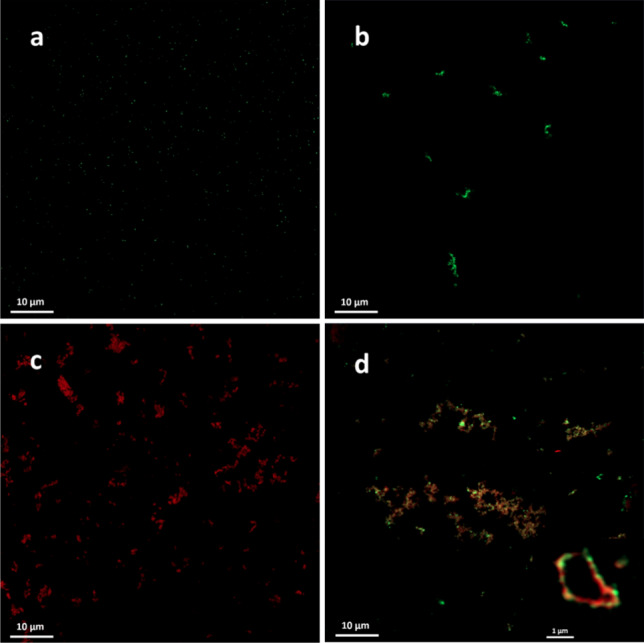
LSCM images of **a** 0.2 mg/ml AqpZ-GFP solubilized in 0.2 w/v% LDAO. **b** 0.2 mg/ml AqpZ-GFP in detergent diluted below the critical micelle concentration (CMC). **c** Atto633 tagged liposomes. **d** Atto633 labeled liposomes with 0.2 mg/ml reconstituted AqpZ-GFP, insert: magnified image showing the structural co-localisation of the two fluorescent channels in a circular pattern, indicating the presence of proteoliposomes

As seen in Fig. 6a, detergent solubilized AqpZ-GFP displayed green fluorescence, visible by the appearance of small, individual green particles. This pattern served as an indication of correctly folded protein in contrast to aggregates, which tend to group together in a “knot-like” manner caused by the deliberate decrease of detergent below its critical micelle concentration (CMC) demonstrated in Fig. 6b (Sung et al. 2015). Liposomes displayed an affinity to cluster together which is clearly visible on the LSCM images, where larger sample areas were imaged, and is also indicated by the close proximity of vesicles on the Cryo-TEM images (Fig. 4, Fig. 6c). This grouping seems to be irrespective of protein reconstitution. Figure 6d displays a clear co-localization of the two channels for the 0.2 mg/ml AqpZ-GFP reconstituted liposomes, with smaller circular shapes visible, corresponding to liposomes. This indicates the formation of proteoliposomes. Furthermore, the insert of Fig. 6d shows a magnified image of one giant unilamellar vesicle (GUV) with several reconstituted proteins. Unfortunately, the resolving power of LSCM limits the resolution of smaller vesicles or the quantification of reconstitution by any means, hence this tool was only used for visualization and qualitative assessment of protein reconstitution.

### Stimulated emission depletion microscopy

STED was carried out in an attempt to push the resolving power of the light microscopy techniques to super resolution imaging as well as to evaluate the effect of covalent AqpZ labeling post-purification. Owing to compatibility with the method, the selected AqpZ protein tag was the Atto594 NHS-ester dye, which has an absorbance at λ_abs_ = 606 nm (orange) and emission at λ_fl_ = 626 nm (red). The lipid dye was kept the same as for the LSCM method, Atto633-PPE.

**Fig. 7:**
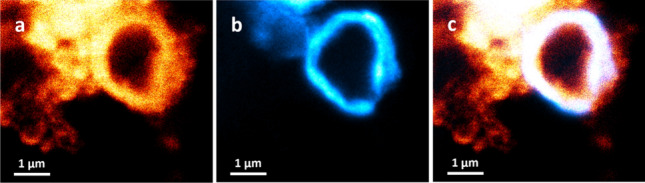
STED images of a GUV labeled with Atto633 and containing AqpZ-Atto594, showing the co-localization of lipid and protein dyes, which signals the insertion of modified AqpZ-Atto594 into the liposome, where **a** displays the Atto366-PPE lipid signal, **b** the Atto594-NHS ester protein signal and **c** the overlay of the two

Figure 7 shows the image of a GUV, first displaying the signal from lipid tagging via the channel for the Atto633 lipid dye (a), followed by the signal from the Atto594 tagged AqpZ (b) and finally the merged signal from both channels (c). As seen from this image, the signals from the lipid and protein dyes seemed to co-localize along the bilayer area of the GUV, which indicates that the tagged protein embedded into the liposome membrane. This result demonstrates that the covalent labeling reaction with the Atto594 did not affect the reconstitution ability of AqpZ. For more information, including the STED images of empty liposome controls please see Supplementary Fig. 3.

### Stopped flow-light scattering

To verify that the reconstituted AqpZ retains its functionality once incorporated into the liposomes, SF-LS was carried out on extruded liposomes to test the permeability of the different vesicle systems.

**Fig. 8:**
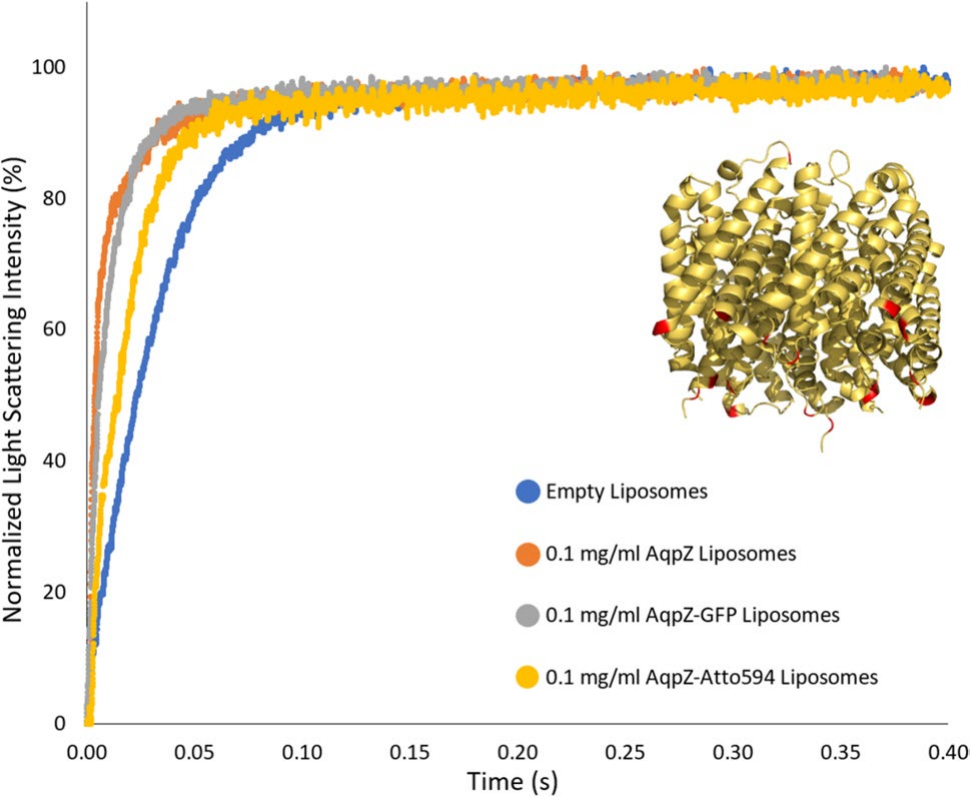
Normalized SF-LS data of empty liposomes as well as liposomes containing 0.1 mg/ml of AqpZ, AqpZ-GFP or Aqp-Atto594, exposed to a 0.5 M NaCl solution. Insert displaying the tilted side view structure of the AqpZ tetramer, with its lysine residues highlighted in red color. The model was generated using PyMol 2.5.2 and the AqpZ PDB structure, 2ABM. (Kim et al. 2020)

Figure 8 and Table 2 show the normalized light scattering data and K values for empty liposomes as well as three proteoliposome samples reconstituted with a variant of AqpZ at 0.1 mg/ml protein concentration. As visible, all AqpZ containing vesicles showed an increase in activity, however protein labeling influenced the functionality of AqpZ as both recombinant (AqpZ-GFP) as well as, covalent (AqpZ-Atto594) labeling detrimentally affected the speed of vesicle shrinkage. This is in contrast to the findings of other studies performed with both genetically and covalently tagged AqpZ liposomes, reporting a maintained permeability performance (Sharma et al. 2022; Ren et al. 2017). Although the activity of AqpZ-GFP was only slightly lower compared to AqpZ, in case of labeling with the Atto594-NHS dye, a significant drop within the protein’s functionality was observed. It is not possible to conclude from these experiments alone whether the cause is a steric one from the label or simply reduced reconstitution efficiency compared to unlabeled protein. However, in the former case, the decrease may be due to the labeling chemistry, where the dye attaches to the primary amines present in the protein structure. Besides making up the N-terminus of the polypeptide chain, primary amines are also present in the side chains of lysine residues, giving rise to potential nonspecific binding. Owing to the location of these lysine moieties within the protein structure, binding of the dye can result in the partial blocking of one or more water channels of the AqpZ tetramer, subsequently hindering protein activity (Fig. 8, insert). However, since the degree of labeling (DoL) was rather low with the Atto594 tag (0.3 dye molecules per protein, for DoL calculations see Supplementary Table 2), it is more probable that the labeling reaction itself resulted in the compromised functionality, either by denaturing the protein or by blocking its pores via changes within the protein–detergent micellar interactions. Regardless, the AqpZ sample that underwent covalent labeling with the Atto594 dye had a significant loss in its ability to transport water. Based on the values reported in Table 2, the K value ratio of 4.6 to 2.7 to 1, can be calculated for the AqpZ, AqpZ-GFP and AqpZ-Atto594 reconstituted samples respectively, following correction with the empty liposome control. These results indicate that both recombinant and covalent fluorophore attachment affected AqpZ activity and successful protein reconstitution into liposomes cannot be used alone as a measure of maintained protein functionality. Since untagged AqpZ reconstituted liposomes showed the highest activity, in the following proteoliposomes reconstituted with different concentrations of this AqpZ variant are investigated further.

**Table 2:** Rate constant, K, values with standard deviation for samples described in Fig. 8, extracted from the average of five independent measurements for each sample and fitted against a double exponential function

	K(s^−1^)
Empty liposomes	33.3 ± 0.14
0.1 mg/ml AqpZ Liposomes	153.0 ± 14.25
0.1 mg/ml AqpZ-GFP Liposomes	102.4 ± 5.01
0.1 mg/ml AqpZ-Atto594 Liposomes	59.2 ± 1.49

**Fig. 9:**
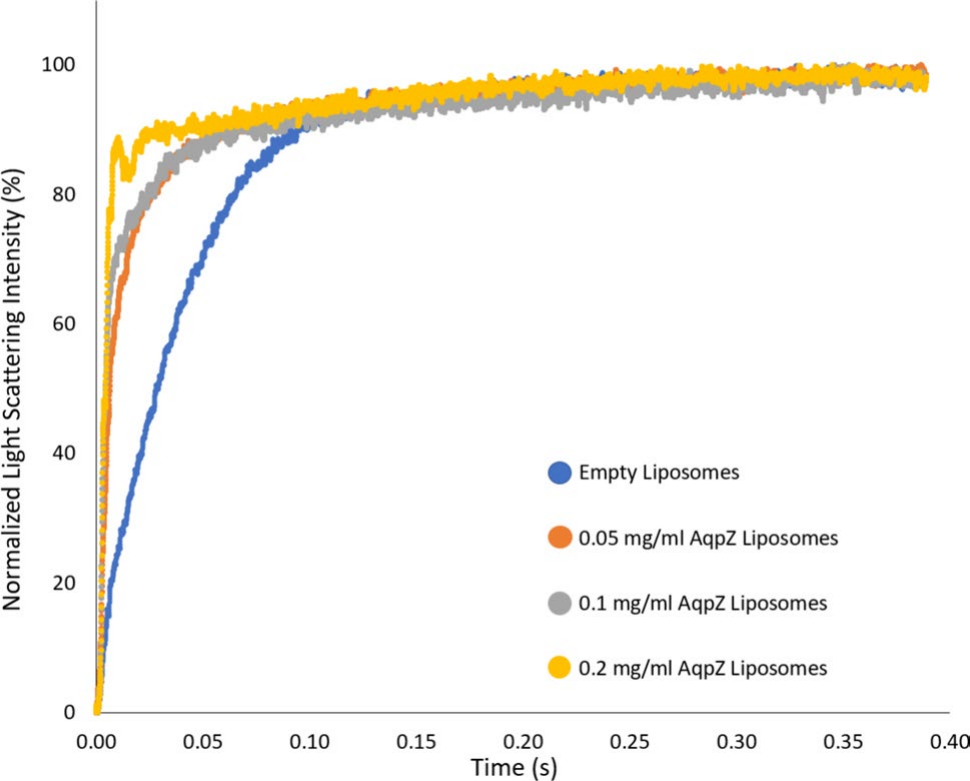
Normalized SF-LS data of empty liposomes as well as AqpZ reconstituted liposomes at three different protein concentrations, exposed to a 0.5 M NaCl solution

Figure 9 displays the normalized light scattering curves for 0.05 mg/ml, 0.1 mg/ml and 0.2 mg/ml AqpZ reconstituted liposomes, showing a clear difference between the four samples, further showcased by the results of its biological duplicate set presented in Supplementary Fig. 4. Osmotic equilibrium was reached the fastest for the sample containing the highest concentration of reconstituted Aqps (yellow) and slowest for the sample containing no Aqps at all (blue), which is in alignment with reported results (Erbakan et al. 2014). An artefact can also be seen for the sample with 0.2 mg/ml AqpZ, which likely indicates an organizational change in the bilayer upon a high concentration of reconstituted protein, but the exact mechanisms underlying this phenomenon are yet to be understood. Table 3 and Supplementary Fig. 5 depict the K values for the different samples, indicating a linearly growing trend following their respective concentrations. K ratios of the 0.05 mg/ml, 0.1 mg/ml and 0.2 mg/ml AqpZ reconstituted liposomes are 1:1.82:3.28, respectively, following correction with the empty liposome control. Based on the K value and using Equation S2, the P_f_ value can be calculated for the bilayer of different samples, which is an important parameter when studying membrane biophysics (for detailed calculations see Supplementary Table 3) (Grzelakowski et al. 2015). As observed in Table 3, values once again increased with the increasing amounts of reconstituted AqpZ protein. Conversion of the derived permeabilities to single channel values are also reported in Table 3 and within experimental error produce almost identical values in close agreement with the previously reported value of p_f_ = 2·10^–14^ cm^3^/s (Pohl et al. 2001).

**Table 3:** Rate constant, K, osmotic permeability, P_f_, values with standard deviation for samples described in Fig. 9 and permeability per AqpZ p_f_ (Wachlmayr et al. 2022). K was extracted from the average of five independent measurements for each sample, fitted against a double exponential function and P_f_ was calculated based on Equation S2 with data presented in Table 1. For detailed calculations, see Supplementary Table 3

	K (s^−1^)	P_f_ (µm/s)	p_f_ (10^−14^ cm^3^/s)
Empty liposomes	26.7 ± 0.47	6.39 ± 0.11	-
0.05 mg/ml AqpZ Liposomes	96.0 ± 7.41	21.55 ± 1.66	2.48 ± 0.27
0.1 mg/ml AqpZ Liposomes	152.5 ± 13.17	39.23 ± 3.39	2.69 ± 0.28
0.2 mg/ml AqpZ Liposomes	254.1 ± 31.37	62.52 ± 7.72	2.29 ± 0.32

### Small-angle X-ray scattering

SAXS was used to structurally characterize the lipid bilayer and to get an independent, quantitative assessment of the protein insertion efficiency.

**Fig. 10:**
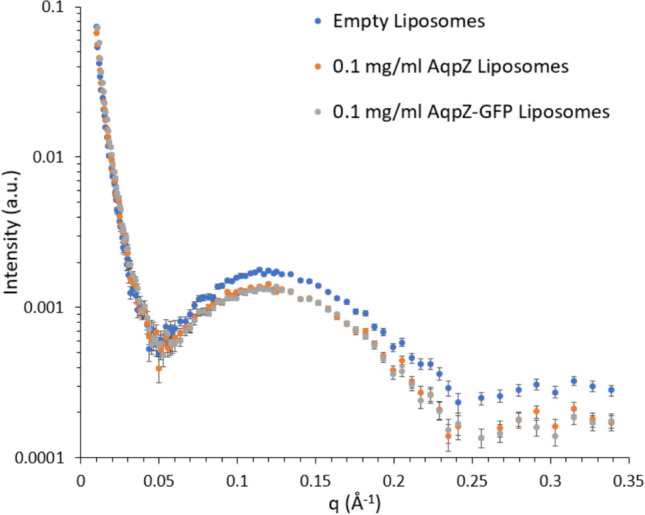
Background subtracted, re-scaled and logarithmically re-binned SAXS data for empty liposomes as well as AqpZ and AqpZ-GFP containing liposomes at 0.1 mg/ml reconstituted protein concentration

Figure 10 displays the scaled, background subtracted and log-binned SAXS data for liposomes with 0.1 mg/ml reconstituted AqpZ and AqpZ-GFP in 1xPBS buffer as the sample background. The two protein variants were selected as they gave the highest activity profiles when reconstituted in the liposomes, based on the SF-LS data (Fig. 8). While the empty liposomes displayed a distinct scattering curve, the signal for both protein containing samples showed a similar tendency, compared to their empty counterpart. This can be explained by the insertion of AqpZ into the liposome bilayer, causing the replacement of lipid tail and head groups by the protein and solvent, respectively. This process is expected to decrease the contrast of the lipid bilayer to the solvent background, subsequently leading to the observed drop within the scattering intensity. Interestingly, both proteoliposome samples showed the same profile, which was against expectations as the GFP tag was presumed to interfere with the overall sample scattering due to its large size (28 kDa) (Verdonck 2009). It is however possible that the linker present between the AqpZ and GFP sequences, distanced the GFP tag just enough from the AqpZ sequence, to not interfere with its scattering signal.

**Fig. 11:**
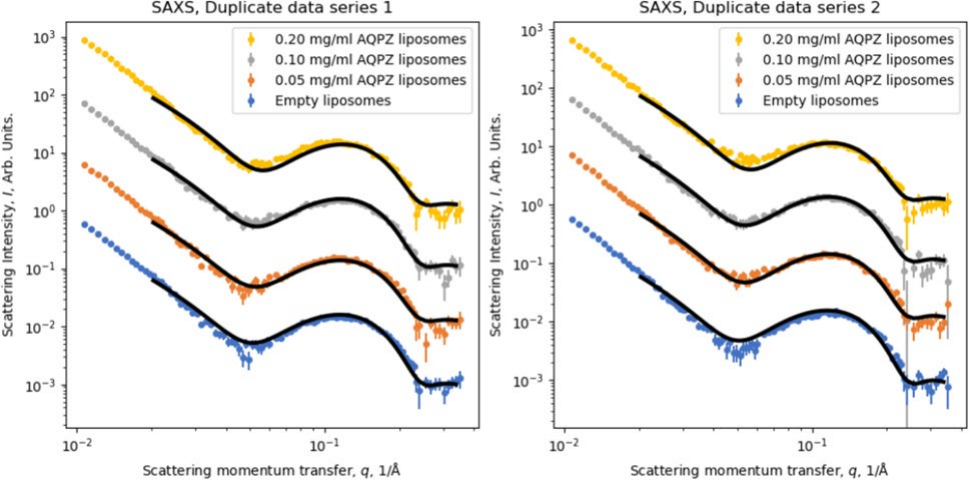
SAXS data and fits using the bilayer model outlined in the text. The individual datasets have been manually scaled for clarity

In order to model AqpZ reconstitution into the liposomes, AqpZ was selected as it does not contain any tags that could interfere with the protein’s insertion efficiency or activity. Figure 11 displays the processed and fitted SAXS data for empty liposomes as well as proteoliposomes reconstituted with varying concentrations of AqpZ in 1xPBS buffer. Samples were non-extruded to ensure the same concentration. To verify the reproducibility of results, the experiment was run with independently prepared sample duplicates, where the first sample set is represented in Fig. 11a and the second in 11b. SAXS was used to gain information on the bilayer characteristics of liposomes with and without different concentrations of reconstituted AqpZ, which can be observed at approximately 0.05 < q < 0.2 Å^−1^. Studying this region, it is expected that when the protein is reconstituted, it replaces a section of the bilayer, therefore the more protein is reconstituted, the less lipids are present in the vesicles and the higher the influence of the scattering properties of the protein on the overall sample scattering profile. To further evaluate the effects of protein insertion on the lipid bilayer and extract quantitative information regarding the different samples with respect to the bilayer area replaced by AqpZ, the datasets were subjected to model fitting.

The refined parameters are presented in Table 4 and are in line with the general expectations. It can be noted that the dimensions of the hydrophobic bilayer region generally agree with the above presented high magnification Cryo-TEM findings as well as with published values for similar lipid compositions (Grzelakowski et al. 2015). The trend in the AqpZ content of the various samples does increase as expected, indicating that the protein indeed incorporated in the bilayer and is yet to reach a possible saturation limit. However, it is clear that the model and refinement scheme produce considerable error bars on several parameters, in part on the AqpZ fractions as these were only refined from a single dataset in the global refinement scheme.

**Table 4:** Parameters refined from the data presented in Fig. 11 using the model described in Eq. 5. The full set of parameters can be found in the Supplementary Information

Parameter	Refined value, data series 1	Refined value, data series 2
Reduced fitting error, χ^2^	3.54	2.47
AqpZ fraction, x, prep. w. 0.05 mg/ml	0.0094 ± 0.025	0.016 ± 0.027
AqpZ fraction, x, prep. w. 0.1 mg/ml	0.012 ± 0.024	0.017 ± 0.028
AqpZ fraction, x, prep. w. 0.2 mg/ml	0.030 ± 0.032	0.035 ± 0.036
Length of tail, L_t_, Å	14.3 ± 3.7	14.6 ± 2.8
Length of head, L_h_, Å	6.65 ± 3.1	6.85 ± 2.9
Interface roughness, R, Å	5.37 ± 4.8	5.04 ± 5.0

The lamellar model introduced in the Methods section appeared to be able to reproduce the overall features in the data, even in the more constrained global model refinement setting. As the model was iteratively developed, it can be reported that the inclusion of a small contribution of micelles (representing some lipid aggregates, as previously observed in the ultracentrifugation experiments, for results see Supplementary Information and Supplementary Fig. 2) was important to capture the shape of the data around q = 0.05 Å^−1^ and that adding interface roughness to the model improved the overlap between the fit and data for q > 0.2 Å^−1^. The latter is attributed to the fact that the geometric model did not attempt to capture the finer structure of the lipid membrane and did not incorporate phenomena such as thickness fluctuations and curvature. One shortcoming of the model is its ability to capture the low-q behavior. Combining the bilayer model with an overall vesicle model was attempted, but the low-q scaling was not correct. A possible explanation is a small population of larger aggregates of some form which was not accounted for, however there is no immediate evidence from microscopy what these structures might be.

## Concluding remarks

Overall, the combined results of this work demonstrated the successful insertion of the AqpZ protein within the bilayer of soy PC liposomes. The selected approach combined findings from DLS, Cryo-TEM, LSCM, STED, SF-LS and SAXS to provide an in-depth structural and functional assessment. Light microscopy analysis indicated the successful incorporation of fluorescently labeled AqpZ variants into liposomes. However, while studying protein functionality further, it was revealed that the attachment of recombinant and covalent protein labels detrimentally influenced the water flow of AqpZ, compared to the untagged variant. To demonstrate protein reconstitution quantitatively, a concentration series of proteoliposomes was subjected to SF-LS analysis, indicating a linear increase of the rate constant and osmotic permeability values with respect to the amount of reconstituted protein and without a saturation limit being reached. Conversion of the derived permeabilies to single channel values produced identical values within experimental error in agreement with previously reported values supporting the conclusion of full insertion of the untagged AqpZ. Given the identical scattering patterns of AqpZ and AqpZ-GFP (Fig. 10) it is tentatively concluded that the tagged variant is also fully inserted, however, it is unclear if some structural arrangement, not detectable by SAXS, could in principle account for the reduced water flow of those assemblies.

Findings of the microscopy techniques indicating a structural protein reconstitution were therefore in agreement with the increased water transport profile of AqpZ containing liposomes. A novel SAXS analysis showed a general agreement with the permeability results, where the refined values for the smallest AqpZ concentration posed a degree of uncertainty as the effect of protein insertion on the characteristics of the bilayer was the smallest in that case. Regardless, the global fitting method enabled the extraction of solid quantitative estimates with respect to the amount of protein inserted into the membrane. It is anticipated for this strategy to find increased use for similar characterization scenarios and with the aid of superior data quality from synchrotron experiments, increased sensitivity should be achievable. All in all, the presented work demonstrated an analytical approach for the study of AqpZ incorporated proteoliposomes both on a structural and functional level. Findings are expected to contribute to the development of improved reconstitution assemblies that hold a high importance for biomimetic membrane applications.

## Supplementary Information

Below is the link to the electronic supplementary material.

Supplementary file1 (DOCX 1471 KB)

## Data Availability

Data are available from the corresponding author upon reasonable request.
