# Supplementary material for: Characterization of Aquaporin Z proteoliposome structure and functionality via microscopy and scattering methods
Source: Eur Biophys J. 2025 Aug 7;54(7):463–76. doi: 10.1007/s00249-025-01790-8 (PMC12640340; doi:10.1007/s00249-025-01790-8)
Supplement: Supplementary file 1 — Supplementary file1 (DOCX 1471 KB) [file 249_2025_1790_MOESM1_ESM.docx]

Supplementary Information

Protein sequences

The amino acid residue sequences of the proteins presented in this study, including recombinantly expressed labels.

Highlighted in red: Leu-Glu linker
Highlighted in yellow: His tag
Highlighted in green: Green Fluorescent Protein (GFP) tag
Highlighted in blue: Tobacco Etch Virus (TEV) cleavage site

AqpZ (corresponding to Protein Data Bank (PDB) structure, 2ABM)

MFRKLAAECFGTFWLVFGGCGSAVLAAGFPELGIGFAGVAAFGLTVLTMAFAVGHISGGHFNPAVTIGLWAGGRFPAKEVVGYVIAQVVGGIVAAALLYLIASGKTGFDAAASGFASNGYGEHSPGGYSMLSALVVELVLSAGFLLVIHGATDKFAPAGFAPIAIGLALTLIHLISIPVTNTSVNPARSTAVAIFQGGWALEQLWFFWVVPIVGGIIGGLIYRTLLEKRD

AqpZ-His

MFRKLAAECFGTFWLVFGGCGSAVLAAGFPELGIGFAGVAAFGLTVLTMAFAVGHISGGHFNPAVTIGLWAGGRFPAKEVVGYVIAQVVGGIVAAALLYLIASGKTGFDAAASGFASNGYGEHSPGGYSMLSALVVELVLSAGFLLVIHGATDKFAPAGFAPIAIGLALTLIHLISIPVTNTSVNPARSTAVAIFQGGWALEQLWFFWVVPIVGGIIGGLIYRTLLEKRDLEHHHHHHHHHH

AqpZ-GFP-His

MFRKLAAECFGTFWLVFGGCGSAVLAAGFPELGIGFAGVALAFGLTVLTMAFAVGHISGGHFNPAVTIGLWAGGRFPAKEVVGYVIAQVVGGIVAAALLYLIASGKTGFDAAASGFASNGYGEHSPGGYSMLSALVVELVLSAGFLLVIHGATDKFAPAGFAPIAIGLALTLIHLISIPVTNTSVNPARSTAVAIFQGGWALEQLWFFWVVPIVGGIIGGLIYRTLLEKRDENLYFQ↓SQFMRKGEELFTGVVPILVELDGDVNGHKFSVSGEGEGDATYGKLTLKFICTTGKLPVPWPTLVTTFGYGVQCFARYPDHMKRHDFFKSAMPEGYVQERTIFFKDDGNYKTRAEVKFEGDTLVNRIELKGIDFKEDGNILGHKLEYNYNSHNVYIMADKQKNGIKVNFKIRHNIEDGSVQLADHYQQNTPIGDGPVLLPDNHYLSTQSALSKDPNEKRDHMVLLEFVTAAGITHGMDELYKHHHHHHHHHH

**Sodium dodecyl sulfate-polyacrylamide gel electrophoresis (SDS-PAGE) result of AqpZ-Atto594**

Verification of the AqpZ labeling reaction with the Atto594 dye via SDS-PAGE.

Supplementary Table 1: Sample overview of the SDS-PAGE gel.

| Lane Nr. | Sample |
| --- | --- |
| 1 | PageRuler™ prestained protein ladder (Thermo Fisher Scientific, USA) |
| 2-7 | Size exclusion chromatography (SEC) peak fractions of the AqpZ-Atto594 labeled protein |
| 8 | Unlabeled AqpZ (Positive Control) |


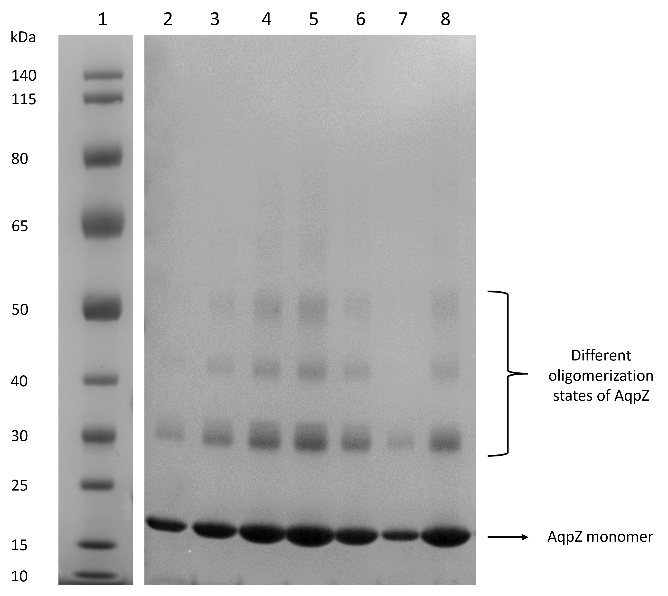


Supplementary Figure 1: SDS-PAGE gel visualization of (lanes 2-7) peak SEC fractions after the labeling reaction of AqpZ with the Atto594 NHS-ester dye, (lane 1) the molecular weight marker and (lane 8) the AqpZ untagged protein as a positive control.

**Calculation of the degree of AqpZ labeling (DoL) with the Atto594 NHS-ester dye**

The DoL for AqpZ with the Atto594 dye can be calculated based on absorbance measurements and the following equation provided by the dye manufacturer, Atto-Tec (Germany):

$$DoL= \frac{A_{max}/\varepsilon_{max}}{A_{prot}/\varepsilon_{prot}}=\frac{A_{max}\cdot\varepsilon_{prot}}{(A_{280}-A_{max}\cdot{CF}_{280})\cdot\varepsilon_{max}} (S1)$$

where, A_max_ is the absorbance of the Atto594 dye at its absorption maximum, ε_max_ is the extinction coefficient of the Atto594 dye at its absorption maximum, A_prot_ is the absorbance of the pure AqpZ protein at its absorption maximum, ε_prot_ is the extinction coefficient of the AqpZ protein at its absorption maximum, A_280_ is the measured absorbance of the Atto594 labeled protein sample and CF_280_ is the correction factor for the absorbance of the Atto594 dye.

The below table showcases the exact values used in combination with Equation S1, to calculate the DoL for AqpZ-Atto594:

Supplementary Table 2: Calculation sheet for the degree of AqpZ labeling with the Atto594 NHS-ester dye, based on the values specified in Equation S1.

|  | A_max_ | ε_prot_ (M^-1^ cm^-1^) | A_280_ | CF_280_ | ε_max_ (M^-1^ cm^-1^) | **DOL** |
| --- | --- | --- | --- | --- | --- | --- |
| AqpZ-Atto594 | 0.3505 | 35075 | 0.5119 | 0.5 | 120000 | **0.304** |

Calculation of osmotic permeability, P_f_

The P_f_ value can be calculated based on the stopped flow-light scattering (SF-LS) rate constant, K, measurements and the following equation, with the detailed values found in Supplementary Table 3. Furthermore, the molar volume of water, V_w,_ has the value of 0.018 L/mol and the osmolarity difference, Δ_osm,_ between 1xPBS and 0.5 M NaCl is 0.6 Osmol/L. Note that for the surface area and volume calculations, liposomes were assumed to be perfect spheres.

$$\boldsymbol{P}_{\boldsymbol{f}}\boldsymbol{=}\frac{\boldsymbol{K}}{\left( \boldsymbol{S}_{\boldsymbol{0}}\boldsymbol{/}\boldsymbol{V}_{\boldsymbol{0}} \right)\boldsymbol{\cdot}\boldsymbol{V}_{\boldsymbol{w}}\boldsymbol{\cdot}\boldsymbol{\Delta}_{\boldsymbol{osm}}}\boldsymbol{(S}\boldsymbol{2)}$$

Supplementary Table 3: Osmotic permeability values for liposome samples without reconstituted AqpZ and with three different concentrations of reconstituted AqpZ.

|  | Empty Liposomes | 0.05 mg/ml AqpZ  Liposomes | 0.1 mg/ml AqpZ  Liposomes | 0.2 mg/ml AqpZ Liposomes |
| --- | --- | --- | --- | --- |
| K (initial rate constant, s^-1^) | 26.7 | 96.0 | 152.5 | 254.1 |
| Z-avg (average diameter, nm) | 132.9 | 124.7 | 142.9 | 136.7 |
| P_f_ (osmotic permeability, µm/s) | 6.39 | 21.55 | 39.23 | 62.52 |

**Ultracentrifugation separation of liposomes from lipid aggregates**

Ultracentrifugation study was performed for the separation of liposomes from other lipid aggregates. Supplementary Figure 2 displays the experimental overview as well as the results of the experiment, analyzed via fluorescent light microscopy.


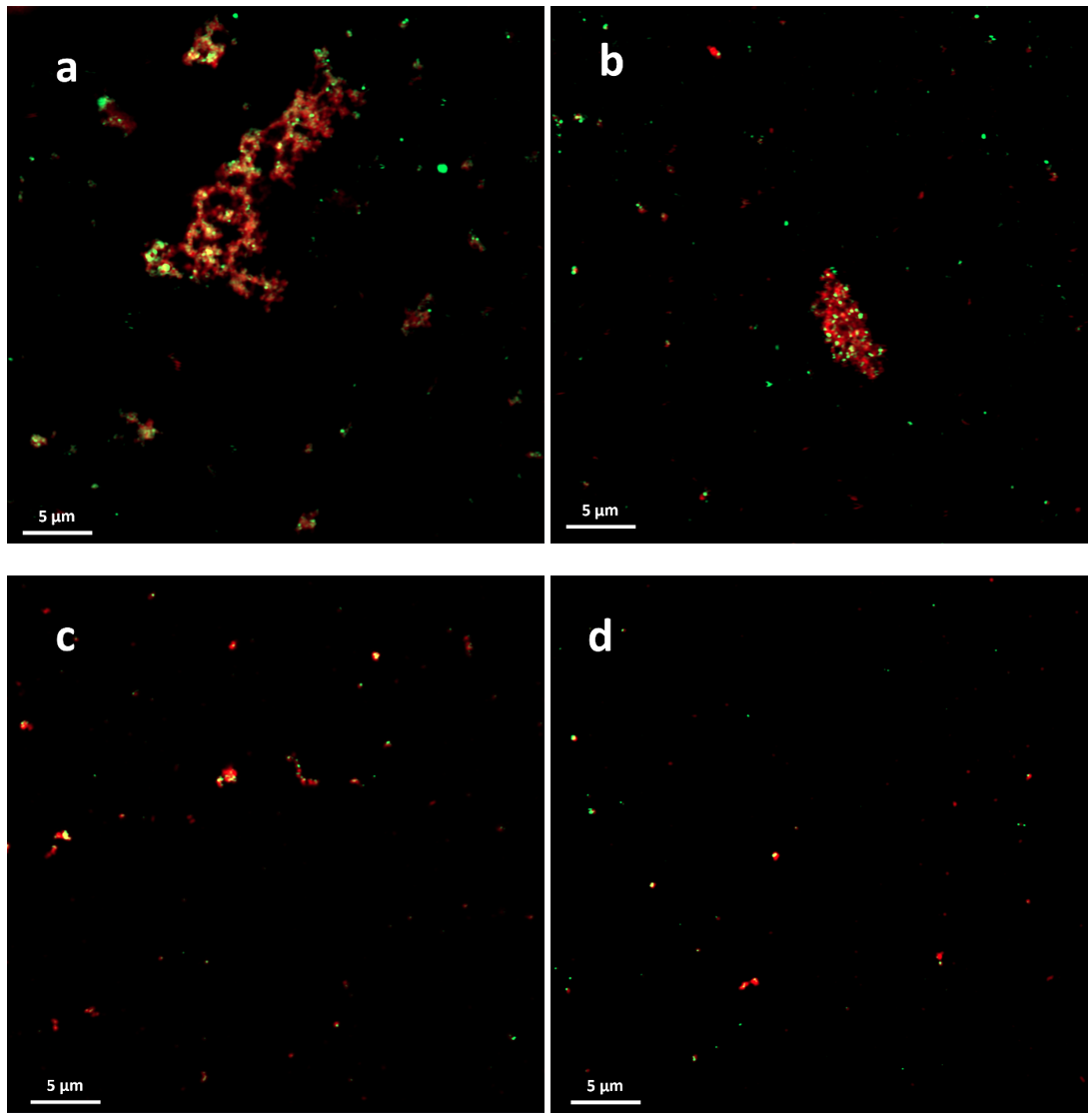


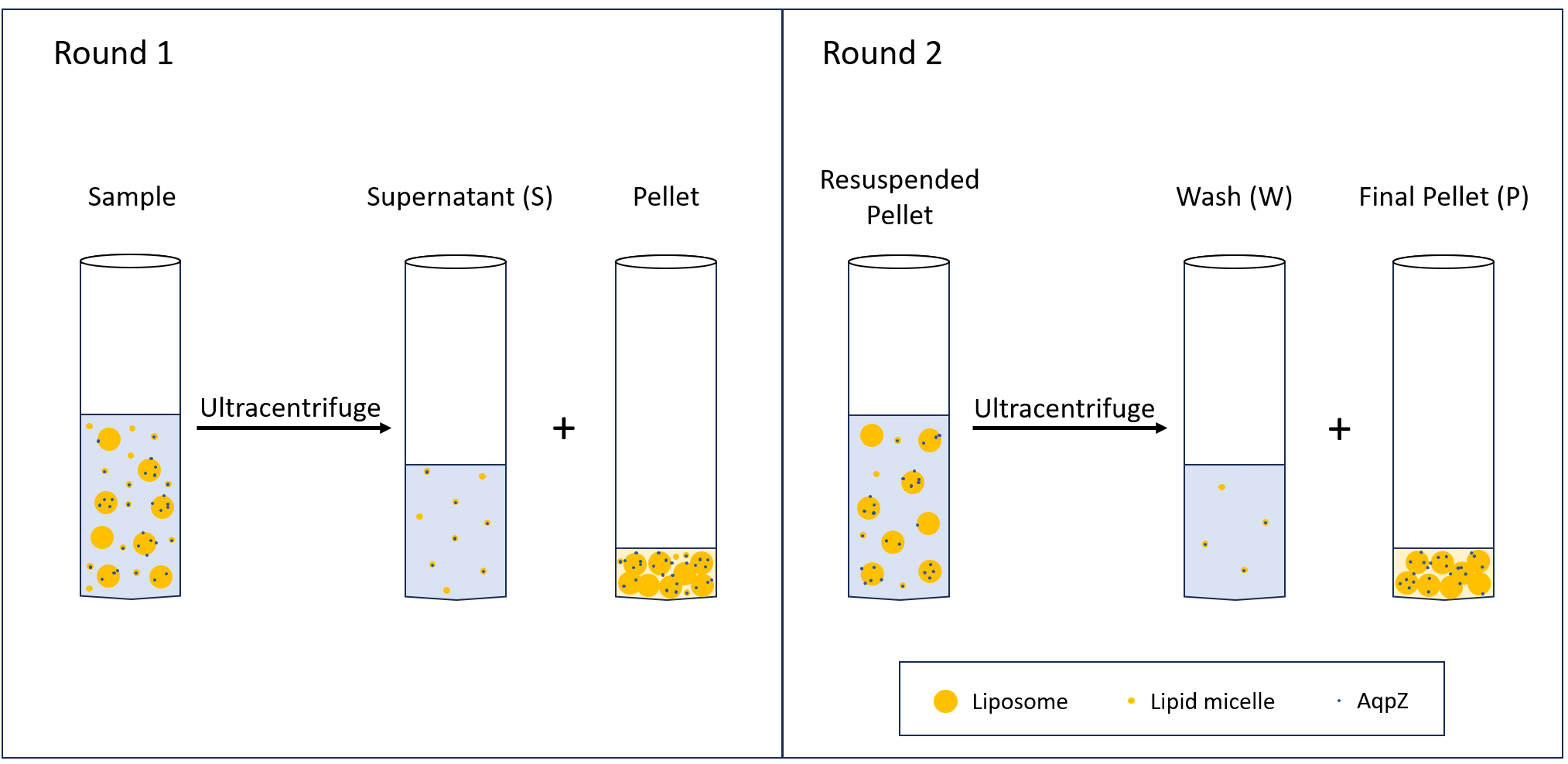


Supplementary Figure 2: Ultracentrifugation study showing (left) the schematics of the two-round ultracentrifugation experiment for the separation of AqpZ containing lipid aggregates from vesicles and (right) the result of the laser scanning confocal microscopy (LSCM) analysis of the different fractions obtained during ultracentrifugation, showing (a) the sample before ultracentrifugation, (b) the P fraction, (c) the S fraction and (d) the W fraction.

**STED images of no-protein control liposomes vs liposomes containing the AqpZ protein**

The following STED images accompany the results presented in Figure 7. The first set of images display the STED signal of empty control liposomes without added protein, solely containing the Atto633 lipid label. As seen the Atto594 channel appears to show no signal. The second set of images showcase the Atto633 labelled liposomes containing the AqpZ-Atto594 protein. Here both the protein and lipid signal appear brightly and show a clear co-localization on the merged image, indicating the specificity of the Atto594 signal towards protein presence.


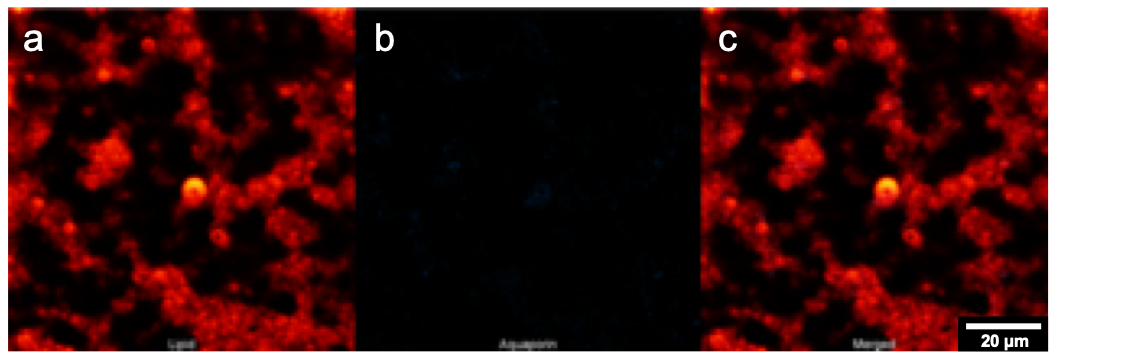


**
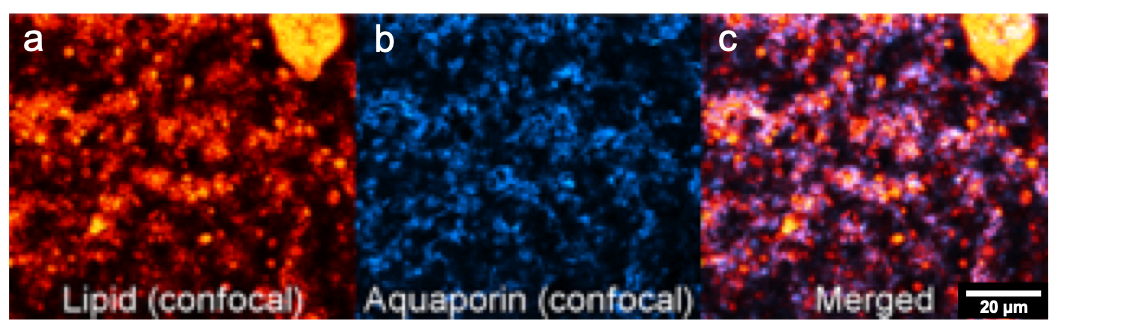
**

Supplementary Figure 3: STED images of liposomes, where (a) displays the Atto633 (lipid tag) channel (b) the Atto594 (protein tag) channel and (c) the two channels merged. The top row corresponds to the empty control liposome sample and the bottom row to the proteoliposome sample.

**SF-LS data presented for the biological duplicate sample set of the liposomes containing the AqpZ concentration series**

**
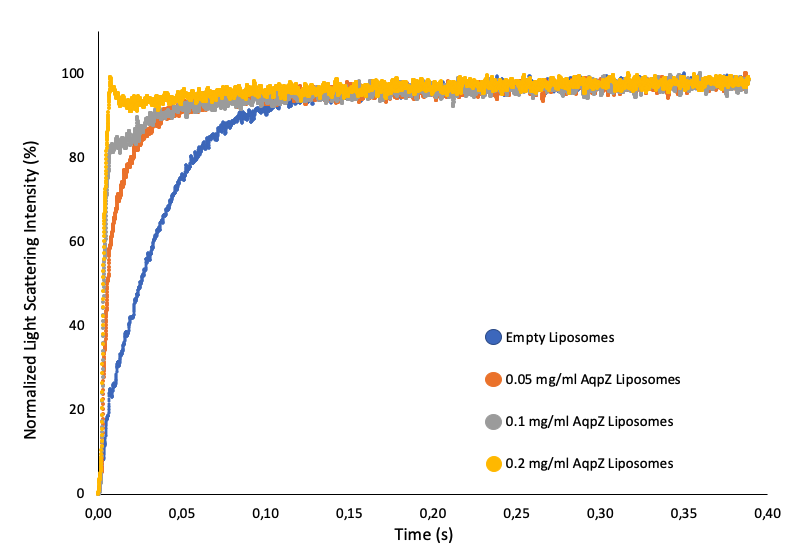
**Biological duplicate set corresponding to the samples presented in Figure 9. As seen, the normalized light scattering signal displays the same tendency for both duplicate sets, where the osmotic equilibrium was reached first for the sample containing the highest concentration of reconstituted protein (yellow) and last for the sample without any added protein (blue). A similar artefact as seen for the yellow curve of Figure 9 can also be observed here, showcasing a reproducibly appearing phenomenon, which is still not fully understood.

Supplementary Figure 4: Normalized SF-LS data of the biological duplicate set for empty liposomes as well as AqpZ reconstituted liposomes at three different protein concentrations, exposed to a 0.5 M NaCl solution.

**Rate constant, K, values displayed for a concentration series of AqpZ reconstituted proteoliposomes**

Rate constant, K, data shown for empty liposomes as well as 0.05, 0.1 and 0.2 mg/ml AqpZ containing proteoliposomes, corresponding to the data presented in Figure 9.


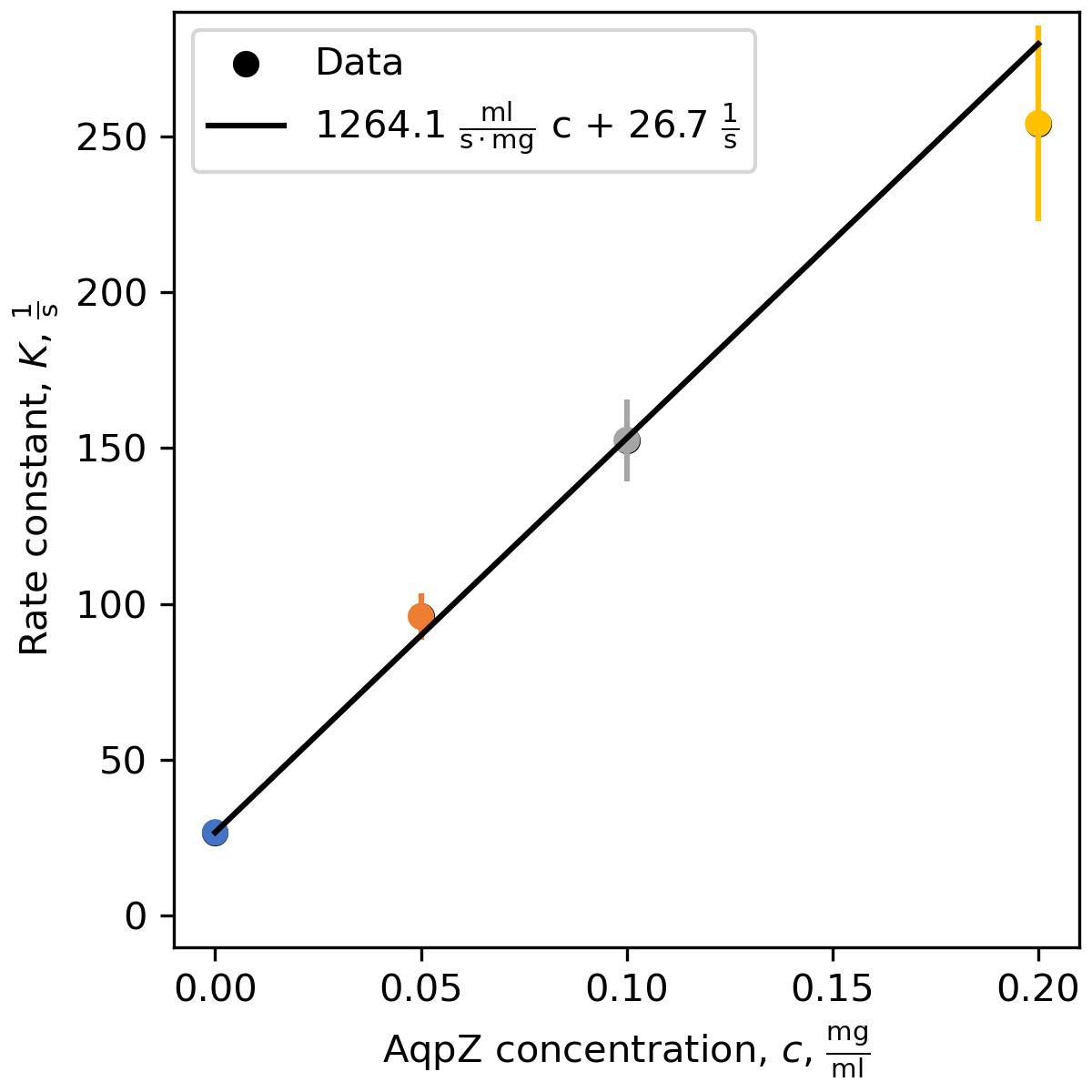


Supplementary Figure 5: Linear trend shown between the rate constant, K, values and AqpZ concentrations of proteoliposomes, with an offset accounting for the empty liposome bilayer.

**Additional information on the SAXS model**

The scattering intensity of the micelle model used in Equation 5 is calculated as follows:

$${I(q)}_{mc}=\left( \frac{4}{3} \pi L_{t}^{3} \left( \Delta\rho_{t}-\Delta\rho_{h} \right) F\left( q L_{t} \right)+ \frac{4}{3} \pi\left( L_{t}+L_{h} \right)^{3} \Delta\rho_{h} F\left( q {(L}_{t}+L_{h}) \right) \right)^{2}$$

where F(…) is the form factor of a sphere,

$$F\left( qR \right)=3\frac{\sin qR-qR\cos qR}{{(qR)}^{3}}$$

It can be noted that while the refinement scheme produces models consistent across all of the data sets, it has the unfortunate consequence that the refinement errors are overestimated on parameters only associated to single data sets (i.e. the aquaporin content, the background, and the proteoliposome scale) as a consequence of the total χ2 across all four data sets not being noticeably impacted by changes to these parameters. However, as seen in Supplementary Table 4, the parameters refined from all data sets simultaneously display sensible refinement errors.

Supplementary Table 4: Full set of SAXS parameters including the values presented in Table 4.

| Parameter | Refined value, Data series 1 | Refined value, Data series 2 |
| --- | --- | --- |
| Reduced fitting error, χ^2^ | 3.54 | 2.47 |
| AqpZ fraction, x, prep. w. 0.05 mg/ml | 0.0094±0.025 | 0.016±0.027 |
| AqpZ fraction, x, prep. w. 0.1 mg/ml | 0.012±0.024 | 0.017±0.028 |
| AqpZ fraction, x, prep. w. 0.2 mg/ml | 0.030±0.032 | 0.035±0.036 |
| Length of tail, L_t_, Å | 14.3±3.7 | 14.6±2.8 |
| Length of head, L_h_, Å | 6.65±3.1 | 6.85±2.9 |
| Interface roughness, R, Å | 5.37±4.8 | 5.04±5.0 |
| Background, B, prep. w. 0 mg/ml, 1/cm | 0.00091±0.0015 | 0.00080±0.0015 |
| Background, B, prep. w. 0.05 mg/ml, 1/cm | 0.00119±0.0015 | 0.00108±0.0015 |
| Background, B, prep. w. 0.1 mg/ml, 1/cm | 0.00101±0.0014 | 0.00100±0.0015 |
| Background, B, prep. w. 0.2 mg/ml, 1/cm | 0.00118±0.0015 | 0.00115±0.0014 |
| Proteoliposome scale, C1, prep. w. 0 mg/ml, 10^-6^ 1/cm^3^ | 3.05±6.6 | 3.77±10.9 |
| Proteoliposome scale, C1, prep. w. 0.05 mg/ml, 10^-6^ 1/cm^3^ | 2.66±5.7 | 3.61±10.2 |
| Proteoliposome scale, C1, prep. w. 0.1 mg/ml, 10^-6^ 1/cm^3^ | 3.17±6.8 | 3.45±9.8 |
| Proteoliposome scale, C1, prep. w. 0.2 mg/ml, 10^-6^ 1/cm^3^ | 2.93±6.2 | 3.00±8.4 |
| Micelle scale, C2, 10^-12^1/cm^3^ | 2.61±6.1 | 2.96±9.3 |
